# Social emotional ability development (SEAD): An integrated model of practical emotion-based competencies

**DOI:** 10.1007/s11031-021-09922-1

**Published:** 2022-01-09

**Authors:** Victor W. Harris, Jonathan Anderson, Brian Visconti

**Affiliations:** University of Florida, Gainesville, USA

**Keywords:** Social emotional ability, Emotional intelligence, Emotional regulation, Empathy, Social intelligence, Sympathy

## Abstract

Social emotional abilities (i.e., specific skills), defined as the set of cognitive abilities, emotion-based knowledge, and behavioral competencies (i.e., skill levels) that facilitate adaptively employing prosocial processes and behaviors (i.e., “actions”), such as emotional regulation and sympathetic and empathetic response behaviors, is contemporarily modeled and measured as emotional intelligence. This conceptualization can be problematic, however, as the two concepts are not the same and traditional methods of measuring emotional intelligence can have limited practical utility. The social emotional ability development (SEAD) theoretical model introduced in this treatise represents a pragmatic and simplified approach to the development of social emotional ability and competency as abstracted from constructs of emotional intelligence, social intelligence, and sociocultural learning theory. Further, the SEAD model reaches beyond the individual as the unit of analysis to explore, conceptualize, differentiate, investigate, and define the hierarchal, bi-directional, and contextual nature of the dimensions of social emotional ability within close relationships. Implications for how the SEAD model can be used by researchers, practitioners, educators, individuals, families, and couples across a broad spectrum of domains and interventions are discussed.

The acquisition and development of emotion-based social abilities, competencies, and behaviors have their foundations in emotional intelligence (Brackett et al., 2016; Mayer et al., 2016), social intelligence (Cantor & Kihlstrom, 1985; Conzelmann et al., 2013), and social emotional learning (Durlak et al., 2015; Wilson-Mendenhall & Barslou, 2016), which begin in infancy and continue across the lifespan. These abilities, competencies, and behaviors are categorized herein as social emotional ability—defined as the set of cognitive abilities (i.e., specific skills), emotion-based knowledge, and behavioral competencies (i.e., skill levels) that facilitate adaptive deployment of prosocial behaviors (i.e., “actions”), such as emotional regulation and sympathetic and empathetic response behaviors. More specifically, social emotional ability is conceptualized in this treatise as intrapersonal and interpersonal abilities and competencies that predict adaptive social behaviors and motivate prosocial actions that lead to increased life-satisfaction and well-being (Batson & Powell, 2003; Spinrad & Eisenberg, 2017). Thus, sympathy and empathy are referred to herein as separate internal abilities and competencies while sympathetic and empathetic response refers to the behavioral actions, such as prosociality (e.g., helping, sharing, comforting, cooperation), these internal abilities and competencies are thought to motivate and predict.

## Developmental, hierarchical, and bidirectional underpinnings of social emotional ability

Social emotional ability is critical to human happiness due to its influence on the quality of social engagement, particularly in close relationships, and the impact it has on decision-making, subsequent life satisfaction, and well-being (Mayer et al., 2016; Rohrer et al., 2018). Maslow, in his seminal work *Hierarchy of Needs* (1954), asserted that social engagement, or connectedness, is a basic human need. A growing body of research has shown that social engagement is an important predictor of life satisfaction and well-being via the developmental, hierarchical, and bidirectional pathways by which human needs are identified and met through social and emotional interactions, decision-making, and problem-solving (Baumeister et al., 2013; Bubolz & Songtag, 2009; Huitt, 2007; Lambert et al., 2010; Rohrer et al., 2018).

## Foundations of social emotional ability: Social and emotional intelligence

Historical and definitional foundations of social emotional ability as abstracted from social and emotional intelligence are tertiarily summarized below.

### Social intelligence

According to Kihlstrom and Cantor, (2011), Thorndike (1920) originally divided intelligence into three general categories: (1) *abstract* (abilities and competencies in *understanding and managing ideas*); (2) *mechanical* (abilities and competencies in *handling concrete* or *physical objects*); and (3) *social* (abilities and competencies in *engaging in “wise” human relations*). Later, according to these authors, researchers provided expanded as well as contrasting definitions of social intelligence abilities and competencies, such as knowledge and understanding of *social matters*, *getting along with people*, *social techniques*, *social stimuli*, *moods*, and *personality traits* (Vernon, 1933), or simply of *general intelligence applied to the comprehension of social situations* (Wechsler, 1958).

Zirkel’s (2000) review of the history and conceptualization of social intelligence added the notion that behavior is a product of its *functionality* and *adaptability*. Zirkel then traced the development of social intelligence from Kelly’s (1955) personality theory, which emphasized that social behavior is a product of cognitive processes, including awareness and understanding of the contextual social world to Rotter’s (1966, 1975) emphasis on perceptively answering the question: “What does the *individual* see as possible for *himself* or *herself* in this situation, and how can *that* help me to understand him or her?” (p. 5). In sum, according to Zirkel (2000),

Social intelligence can be described as a model of personality and individual behavior in which people are presumed to be knowledgeable about themselves and the social world in which they live. Individuals actively use this knowledge to manage their emotions and direct their behavior toward desired outcomes. (p. 20)

Major concepts of the social intelligence theoretical model include that socially intelligent behavior is purposive in at least four ways:

1. *Opportunities* and *risks* are inherent in socially intelligent purposive behaviors, and as a result, they influence the development of cognitions and behavioral decisions to both choose and pursue specific goals;
2. These opportunities, risks, cognitions, behavioral decisions, and their associated goals are highly influenced by *culture*;
3. The development and pursual of these goals are also highly influenced by *self-definition*, *identity*, and the protection of these important *self-conceptions*;
4. Items #1–3 above coalesce to influence “... the strategies individuals use to pursue important goals, regulate their affective experiences, and achieve desired ends in a wide variety of situations.” (Zirkel, 2000, p. 20)

### Emotion and emotional intelligence

Although their origins can be traced to ancient Greece, modern conceptualizations of emotion and emotional intelligence can be more accurately traced from the mid-twentieth century, with the first legitimate measures of emotional intelligence introduced in the early 1990s (Mayer et al., 2000). Emotional intelligence can be conceptualized as the mental ability to activate the *emotional response system* which “coordinates physiological, perceptual, experiential, cognitive, and other changes into experiences of moods and feelings, such as happiness, anger, sadness, and surprise (Smith & Lazarus, 1990, p. 610)” (Mayer et al., 2000, p. 323).

More specifically, Mayer et al. (2008a, b) define *emotion* as “an integrated feeling state involving physiological changes, motor-preparedness, cognitions about action, and inner experiences that emerges from an appraisal of the self or situation” and *emotional intelligence* as “a mental ability (or set of emotional abilities) that permit the recognition, learning, memory for, and capacity to reason about a particular form of information, such as verbal information” (pp. 508–509; see also Kensinger & Schacter, 2016). For the purposes of this theoretical treatise, emotional intelligence is considered “the ability [whether inherited or learned] to monitor one’s own and other’s emotions, to discriminate among them, and to use the information to guide one’s thinking and actions” (Salovey & Mayer, 1990, p. 189).

### Goal and specific abilities of emotional intelligence

The underlying goal of emotional intelligence is to “improve psychological functioning in real life” (Schulze et al., 2005, p. 24) through the cognitive processes of both *perceiving* and *regulating* emotions (Neubauer & Freudenthaler, 2005), including the utilization, appraisal, and expression of emotion (Salovey & Mayer, 1990). Conceptualized as both *personality trait* and *ability*, emotional intelligence can be better understood through identifying at least four specific ability areas (see Bar-On, 2000; Mayer & Salovey, 1997):

1. *Perception, appraisal, and expression of emotion:* Ability to (a) *identify emotion in one’s physical states, feelings, and thoughts*; (b) *identify emotions in other people, designs, artwork, *etc*. through language, sound, appearance, and behavior*; c) *express emotions accurately and to express needs related to those feelings*; and d) *discriminate between accurate or inaccurate or honest versus dishonest expressions of emotion*.
2. *Emotional facilitation of thinking:* Emotions (a) *prioritize thinking by directing attention to important information*, (b) *are sufficiently vivid and available that they can be generated as aids to judgment and memory concerning feelings*; *Emotional mood swings change the individual’s perspective from optimistic to pessimistic, encouraging consideration of multiple points of view*; *Emotional states differentially encourage specific problem approaches such as when happiness facilitates inductive reasoning and creativity*.
3. *Employing emotional knowledge to the recognition, and analysis, and understanding of emotions:* Ability to (a) *label emotions and recognize relations among words and the emotions themselves, such as the relationship between liking and loving*; (b) *interpret the meanings that emotions convey regarding relationships, such as that sadness often accompanies loss*; (c) *understand complex feelings: simultaneous feelings of love and hate, or blends such as awe as a combination of fear and surprise*; and (d) *recognize likely transitions among emotions, such as the transition from anger to satisfaction, or from anger to shame*.
4. *Reflective regulation of emotions to promote emotional and intellectual growth:* Ability to: (a) *stay open to feelings, both those that are pleasant and those that are unpleasant*; (b) *reflectively disengage or detach from an emotion depending upon its judged informativeness or utility*; (c) *reflectively monitor emotions in relation to oneself or others, such as recognizing how clear, typical, reasonable, or influential they are*; (d) *manage emotion in oneself and others by moderating negative emotions and enhancing pleasant ones, without repressing or exaggerating information they may convey*. (Adapted from Neubauer & Freudenthaler, 2005, p. 37)

### Commonalities between social and emotional intelligence

According to the definitions above, emotional intelligence can be subsumed within the general definition of social intelligence with the mutual goal of *engaging in “wise” intrapersonal and interpersonal human relations*. At least four ways in which emotional intelligence can be subsumed within social intelligence are discussed below:

1. Emotional intelligence mental (knowledge, perceptions, appraisals) and emotional abilities (expressions, facilitative and reflective thinking) can permit the recognition, learning, recall, and capacity to reason about: knowledge of social matters, getting along with people, social techniques, social stimuli, moods, and personality traits.
2. Emotional intelligence, in general, can be applied to emotional management and regulation due to the comprehension (e.g., perception, appraisal, recognition, analysis, and understanding) of social situations.
3. Emotional intelligence is influenced by cognitive perceptions of opportunities, risks, choices, decisions, culture, and self-conceptions which can facilitate or inhibit reflective thinking, including emotional and intellectual growth.
4. Emotional intelligence requires adaptive responses, responses which are associated with critical problem-solving. More specifically, adapting to life’s challenges requires critical thinking and the capacity to find solutions to the problems encountered.

### Distinctions between social and emotional intelligence

As early as 1990, Salovey and Mayer described emotional intelligence as a subset of social intelligence. More recently, numerous studies describe social intelligence and emotional intelligence as similar concepts (Joseph & Newman, 2010; Kihlstrom & Cantor, 2011; Lievens & Chan, 2017), a conception clearly verified by the similarities in their many and varied definitional and conceptual descriptions (Hedlund & Sternberg, 2000). In Gardner’s paradigm, emotional intelligence may be more reflective of his *intrapersonal intelligence* conceptualization with a focus on *personal needs, goals, and mental abilities* while social intelligence may be better conceptualized as *interpersonal intelligence* with the focus on adaptively using these and other abilities to solve real-world social problems (Gardner, 1983; Hedlund & Sternberg, 2000).

For the purposes of this treatise, what separates social intelligence from emotional intelligence generally is the *scope of problem-solving* tasks across a wide variety of social and emotional situations and the *actual behaviors* individuals adaptively deploy to both regulate and engage with emotions and contexts to guide social decision-making and interactions. In other words, as noted by Salovey and Mayer (1990), emotional intelligence represents one subset of a far greater set of socially-related intelligences (Gardner, 1983; Topping et al., 2000). Specifically, what distinguishes emotional intelligence (including its broader definitions (Bar-On, 2000, 2006; Goleman, 1995) from social intelligence is the specificity of the four emotional intelligence cognitive ability areas outlined above and summarized below (Mayer & Salovey, 1997).

1. The cognitive ability to identify specific emotions in physical states, feelings, and thoughts in both oneself and others and in objects and symbols manifest through the five senses, accurately articulate and express needs and associated feelings, and discern between authentic and inauthentic expressions of emotion.
2. The cognitive ability to attend to important stimuli and information, selectively prioritize it, entertain multiple perspectives, and use this information and perspectives to make inductive and creative judgments regarding memory and feelings, including modulating emotions from one state to another.
3. The cognitive ability to deploy emotional knowledge to recognize, analyze, and understand emotions, label emotions using words and distinguish between them, including complex emotions and the transitions between emotions.
4. The cognitive ability to remain open to both pleasant and unpleasant feelings, monitor emotions in oneself and others, regulate emotions by intentionally disengaging from one or more emotions depending upon their utility and helpfulness, and regulate emotions without suppressing or ignoring them.

While these cognitive ability areas can easily be subsumed within the current overall structure of social intelligence, emotional intelligence as outlined by Salovey and Mayer (1990) is meant to represent a higher order construct. Still, definitional confusion exists and that extends to multiple measurement issues.

## Social and emotional intelligence measurement issues

Some researchers remain skeptical that social and emotional intelligence can be separated psychometrically (Bar-On, 2006; Hedlund & Sternberg, 2000). When compared to emotional intelligence, the attempted measurement of social intelligence reveals a longer history (Kihlstrom & Cantor, 2011). Major criticisms which surround the measurement of social intelligence include reliance on self-report, lingering questions regarding whether or not it can be separated from abstract, academic intelligence (e.g., cognitive vs. behavioral), issues of convergent and discriminate validity (Hedlund & Sternberg, 2000); Lievens & Chan, 2017), and whether or not new discoveries in neuroscience can resurrect legitimate interest in social intelligence by reconceptualizing it as a viable and valid construct (Kihlstrom & Cantor, 2011).

Operationalizing social intelligence still remains an issue (Kihlstrom & Cantor, 2011).

Dimensions of social intelligence are so widely and variously defined that it can be difficult to determine construct validity. Terms such as *social awareness*, *social competence*, *self-monitoring*, *social skills*, *emotional intelligence*, and *practical intelligence* are often used interchangeably with social intelligence, thus reducing it to a “catch-all” construct (Lievens & Chan, 2017).

The same issue exists with “emotional intelligence” having become a “buzzword” associated with nearly every intrapersonal capability short of IQ (Goleman, 1995; Hedlund & Sternberg, 2000). Locke (2005) stated that “EI has now become so broad and the components so variegated that no one concept could possibly encompass or integrate all of them, no matter what the concept was called; it is no longer even an intelligible concept” (p. 426). Other criticisms surrounding emotional intelligence include vague and incompatible definitions of the construct, discriminant and criterion validity issues, and questions surrounding interpersonal skills training programs and the actual impact of emotional intelligence as a construct on intrapersonal and interpersonal outcomes (Matthews et al., 2007).

### Two predominant emotional intelligence instruments

Some researchers continue to maintain that measuring emotional intelligence is simply a more concise way to measure social intelligence (Landy, 2005, 2006; Lievens & Chan, 2017; Locke, 2005). Bar-On (2000) and Mayer et al. (2000) have done some of the most extensive work to date with regard to measuring emotional intelligence. Bar-On’s *Emotional Quotient Inventory* (EQ-i) is a mixed-method self-report instrument while the *Mayer, Salovey, & Caruso Emotional Intelligence Test* (MSCEIT) instrument is performance- or ability-based. Strengths and weakness of both instruments are discussed below.

### Strengths and weaknesses of the EQ-i and MSCEIT measures

Sometimes referred to as trait emotional intelligence (Matthews et al., 2007), Bar-On’s EQ-i conceptualizes emotional intelligence as both a heritable personality trait, measured by fifteen facets within five broad factors, and as a learned ability (Lievens & Chan, 2017). Critics of the EQ-i measure highlight its broad application to a variety of constructs not associated with emotional intelligence. For example, The EQ-i also measures constructs typically associated with social intelligence, including factors such as interpersonal skills, problem solving, and flexibility, along with personality traits such as optimism (Hedlund & Sternberg, 2000). Moreover, a majority of the EQ-i constructs can be categorized under the Big Five Personality traits with most of these constructs justifiably being housed under Agreeableness and Emotional Stability (Lievens & Chan, 2017; see De Raad, 2005).

While the MSCEIT has been shown by some to be a valid, reliable measure of emotional intelligence (Mayer et al., 2012; Schulze et al., 2005), meta-analyses show that it shares very little in common with the EQ-i with only a 0.14 correlation among constructs (Lievens & Chan, 2017; see Van Rooy et al., 2005). This finding is problematic in that it underscores the problem of not just construct validity but the growing suspicion and mistrust that emotional intelligence is not psychometrically measurable. Moreover, because the MSCEIT correlates highly with verbal ability (0.30 to 0.40), some critics argue that emotional intelligence should simply be called “emotional knowledge” (Lievens & Chan, 2017; see Zeidner et al., 2004).

The MSCEIT has also been widely criticized because it is relatively time consuming, expensive, and generally lacks practical utility. As an intellectual problem-solving measure, its ability to produce any lasting social or behavioral change has been met with mixed reviews (Locke, 2005; Matthews et al., 2007), particularly with respect to increasing life satisfaction (Dabke, 2014; Ruiz-Aranda et al., 2014).

The MSCEIT findings do not suggest that emotional intelligence is unrelated to life satisfaction in general, however (Urquijo et al., 2016). Di Fabio and Kenny (2016), for example, found when self-report measures of emotional intelligence are conceptualized behaviorally and not through cognitive ability, emotional intelligence is more strongly related to perceptions of life satisfaction. Palmer et al. (2002) also found independent effects of emotional intelligence which helped to explain variance in life satisfaction separate from personality factors.

In addition, Kong et al. (2019) have successfully used neuroimaging, specifically brain network topography, to show that *connectivity strength* is predictive of human behavior and related emotions. More specifically, according to these authors, “Behavioral phenotypes across cognition, personality, and emotion could be predicted by individual-specific network topography with modest accuracy” (p. 2533), which would include specific emotions associated with life-satisfaction and well-being.

Finally, while both the MSCEIT and the EQ-i are explicit in measuring proposed emotional intelligence constructs, implicit emotional intelligence skills, such as decoding nonverbal cues or emotional expressions, are not generally assessed in either measure (Ekman, 2007; Matthews et al., 2007). Regardless of the criticisms documented above, the EQ-i and MSCEIT instruments remain two of the most important pioneering measures for guiding the understanding of social and emotional intelligence.

## Summary and conclusions

Concepts and measures of social and emotional intelligence are clearly intertwined, and perhaps as suggested, emotional intelligence can be subsumed into constructs of social intelligence. Additionally, while the demonstration of social and emotional intelligence may require specific and somewhat similar abilities or competencies, such as the recognition and comprehension of social stimuli, subsequent adaptive intellectual problem-solving, and prosocial interaction behaviors, the research to date remains unclear about exactly how this demonstration occurs. The research is also unclear about how the demonstration of social and emotional intelligence can best be measured and how it results in decision-making that leads not only to prosocial interactions, but quality outcomes such as life satisfaction and well-being.

Ongoing questions surrounding emotional intelligence measurement issues that need to be addressed can be parsed into three general categories (Matthews et al., 2007): (1) Is it an *awareness* of and/or an *aptitude* for solving problems in emotional situations? (2) Is it a *set of learned skills* for managing challenging and emergency situations? 3) Is it an *outcome* demonstrating successful management of emotional encounters? Clearly, the EQ-i and the MSCEIT address these questions differently and yet, as noted previously, both these measures, as well as related social intelligence measures, are foundationally important to the understanding of social and emotional ability development in spite of their weaknesses.

### Situating social emotional ability development between social and emotional intelligence

It is this confusion in the literature, lack of differentiation between social and emotional intelligence, difficulty among predominant instruments to adequately measure and discriminate between social and emotional intelligence and other constructs, along with the absence of broad practical utility among these instruments that provided the impetus for the current theoretical treatise. Specifically, in the current study the authors introduce the social emotional ability development (SEAD) theoretical model, and in a successive study the development of the social emotional ability inventory (SEAI) (forthcoming), in an attempt to integrate and synthesize constructs abstracted from emotional intelligence, social intelligence, and sociocultural learning into a viable and *practical diagnostic tool*. This tool can then be used to study and better understand the dynamics of emotion both intrapersonally and interpersonally. Implications for how the SEAD model, integrated with the SEAI, can be used by researchers, practitioners, educators, individuals, families, and couples across a broad spectrum of domains to develop social emotional abilities and competencies into prosocial behaviors which increase life satisfaction and well-being are discussed below.

## Purpose

The inconsistencies and confusion in the literature regarding the investigation and measurement of social and emotional abilities, competencies, and behaviors warrant the need for an integrated theoretical developmental model (SEAD) and measurement instrument (SEAI) which are founded upon salient dimensions of social emotional ability abstracted from social and emotional intelligence theories and instruments. Both the model and instrument must be: (1) congruently framed within context, such as the environmental context of Vygotsky’s sociocultural theory of learning; (2) cognized from individual, family, and couple or other dyadic units of analysis; and (3) easily deployed for practical utility. Consequently, the primary purpose of this treatise is to expand relevant theory by introducing a practical theoretical SEAD model to address these inconsistencies and confusions through conceptualizing, differentiating, investigating, and defining the hierarchal and bi-directional nature of the various dimensions of social emotional ability. The SEAD model was specifically designed to facilitate the development of the integrated self-report social emotional ability inventory (SEAI), which is capable of diagnostic-level measurement of each of nine discrete dimensions, including the measurement of adaptive prosocial behavioral responses. In sum, the SEAD model was designed to provide a broad and simplified explanation of social emotional ability while the SEAI was developed to provide practical utility in the diagnostic process of identifying strengths and guiding remediation of specific deficiencies in social emotional ability.

### Individual and family foundations of social emotional ability

In the face of changing contemporary redefinitions, the family maintains its position globally as the primary childrearing unit. One of the critical milestones of childrearing is the development of social emotional abilities, which begin in childhood through secure or insecure attachment (Bowlby, 1979; Cassidy, 1999; Lewis, 2016; Mann et al., 2017). Healthy development of social emotional ability includes development of the ability to identify and understand emotions, and the ability to manage and modulate emotional experiences (Hamilton et al., 2016; Widen, 2016). The family social and emotional environment and the enduring bonds that exist between family members,^1^ especially the emotional attachment of children to their primary caretakers, are central to social emotional outcomes (Bowlby, 1979; Darling-Churchill & Lippman, 2016). This is important because social emotional ability is related to the developmental trajectory of children, including educational outcomes and important developmental milestones (Darling-Churchill & Lippman, 2016; Elias & Haynes, 2008).

Furthermore, the range and types of emotions expressed by children are greatly influenced by their contextual emotional interactions with primary caretakers (Cassidy, 1999; Gottman et al., 1997) and the socialized patterns they develop through witnessing the parent couple relationship (Buehler, 2020; Gottman et al., 1997; Karney & Bradbury, 2020; Sassler & Lichter, 2020; Smock & Schwartz, 2020). Thought, emotion, and behavior are intricately intertwined through learned patterns of social interaction (Denham, 2007; Gottman et al., 1997; Morales et al., 2005). The impacts of parental emotional investment and the emotional climate of the family on childhood development are long-lived. Social emotional growth, which begins during infancy, provides the foundation for emerging SEAD and associated relationship quality across the lifespan (DeSteno et al., 2013; Gottman et al., 1997; Morales et al., 2005).

One of the most important childhood developmental tasks is emotional regulation (Denham, 2007; Erickson, 1968; Frankel, et al., 2012; Morris et al., 2007). The development of the competency to regulate emotions is seen as central to social emotional ability and is directly related to parental emotional involvement and family emotional climate (DeSteno et al., 2013; Fischer & Manstead, 2016; Suri & Gross, 2016). Healthy emotional expression, a healthy family emotional environment, and emotional relationships between parent and child characterized by responsiveness, warmth, and affection—combined with age-appropriate learning activities and imitative modeling—have been shown to promote optimal social and emotional development (Cassidy, 1999; Giallo et al., 2014; Gottman et al., 1997; Roggman et al., 2008). This is not the case, however, for many children without the opportunity to grow up in an optimal social and emotional context. It is the development of these social and emotional abilities within the context of childhood and across the lifespan that informs the foundational structure of the SEAD model.

## The social emotional ability development model

The SEAD model presents a system of intellectual and behavioral responses invoked by interactions between intellectual capacity, constructs of emotional intelligence and social intelligence theories, and environmental circumstances and contexts. The SEAD does not address physiological emotional development which is intrinsically biologically (Adolphs & Anderson, 2018; Barrett, 2018; Nikolova et al., 2016) and socially driven (Fischer & Manstead, 2016; Keltner et al., 2016); rather, the SEAD specifically addresses social emotional abilities, competencies, and related response behaviors.

Correspondingly, the SEAD presents a practical approach for understanding the complex processes at play in SEAD in a manner that provides the potential for diagnosing strengths and remediating specific deficiencies through a proposed hierarchical and bidirectional theoretical framework. It is important to note that the SEAD does not attempt to explain all dimensions or sub-dimensions inherent to social emotional ability; rather, the authors abstracted constructs specific to social emotional ability from social intelligence and emotional intelligence theories, narrowly differentiated into nine dimensions with the lowest level of latency possible that could meet the requirements as statistically adequate indicators.

This was intentionally executed so that the theoretical framework of this new model could support integration with a self-report instrument capable of identifying and providing guidance for the remediation of specific within-construct deficiencies in social emotional ability. For example, one of the constructs of the SEAD is *emotional clarity*, which was abstracted from the emotional intelligence construct, *self-awareness*. Self-awareness is a complex concept that has proven difficult to measure through self-report instruments (Ashley & Reiter-Palmon, 2012), yet self-report instruments are often used to measure emotional clarity (Boden et al., 2013). One such widely used instrument, the *Difficulties in Emotion Regulation Scale* (DERS), accurately measures clinically relevant levels of emotional clarity by posing statements such as, *“I am clear about my feelings”* (Gratz & Roemer, 2004; Neumann et al., 2010). However, due to the complexity of the construct, the DERS is not capable of providing diagnostic level measurement, as it measures latent *indicators* of emotional clarity, but not within-construct dimensions.

### Dimensions of the SEAD model

Dimensions of the SEAD abstracted from constructs of emotional intelligence theory include the abilities to identify (Lindquist et al., 2016), understand (Harris et al., 2016), and accept (Gottman et al., 1997) emotions; the abilities to attend to one’s emotions (Hajcak et al., 2016), use emotional messages to support decision-making (Clore & Schiller, 2016; Lempert & Phelps, 2016; Mayer et al., 2004), and adaptively regulate emotions (Mayer et al., 2016; Suri & Gross, 2016). Dimensions of the SEAD abstracted from constructs of social intelligence theory include the abilities to adaptively respond sympathetically and empathetically to others (Rahim, 2014; Srivastava & Das, 2016).

Each dimension of the SEAD model contains both an intrapersonal and an interpersonal function. The authors propose that the development of specific intrapersonal social emotional abilities (e.g., identifying one's own emotions) may inform, support, predict, precede and/or run parallel to the development of social emotional interpersonal abilities (e.g., identifying others' emotions). Thus, interpersonal social emotional abilities can be scaffolded with the development of intrapersonal social abilities both independently and in parallel. Additionally, identifying others' emotions may also emerge in parallel to, or inform, support, or predict the individual's ability to identify their own emotions. Thus, in the interpersonal domain, the authors also propose a similar progression from identifying others' emotions to understanding, accepting, attending to, and co-regulating emotions comparable to the intrapersonal domain. Examples of each dimension and their intrapersonal and interpersonal functions are used to highlight these functions below.

**Fig. 1:**
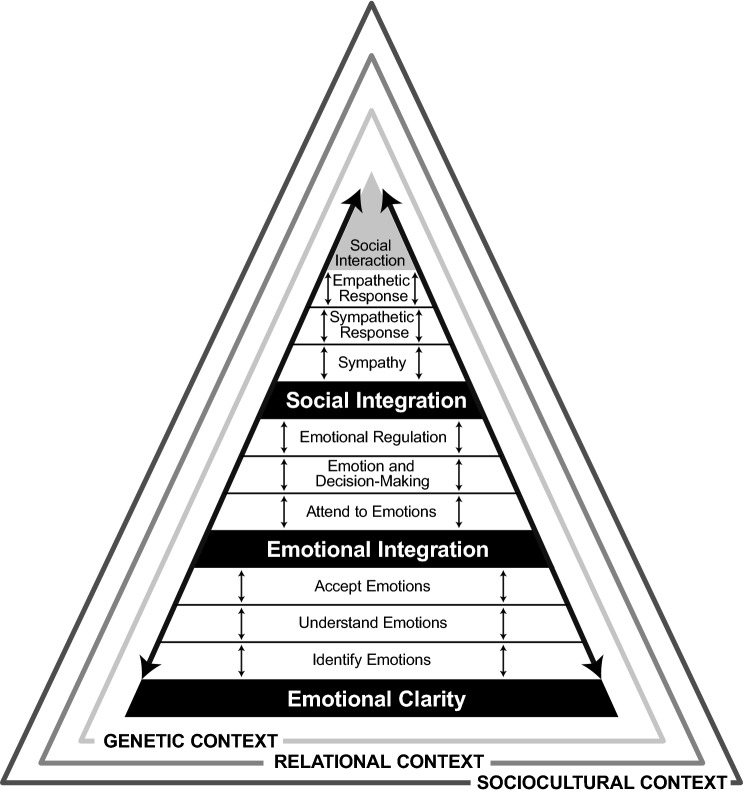
Harris’ social emotional ability development (SEAD) model

The dimensions within the SEAD model are organized into three summative constructs (Fig. 1). The first construct is Emotional Clarity, which is defined as the integration of emotion and thought through the abilities to identify, understand, and accept emotions. The second is Emotional Integration, which is defined as the integration of emotion, thought, and behavior through the abilities to attend to intrapersonal emotions, incorporate emotional messages into decision-making processes, and adaptively regulate emotions. The third is Social Integration, which is defined as the integration of emotion, thought, behavior, and social interaction through the abilities to comprehend the emotional states of others and attend to the interpersonal emotions of others through sympathetic and empathetic social response behaviors.

The SEAD model articulates that higher competency levels of cognitive social emotional abilities, greater levels of competency in emotional regulation behaviors, and sympathetic and empathetic response behaviors, in particular, result in more positive social interactions which lead to increased life satisfaction and well-being (Allemand et al., 2014; Eisenberger, 2016; Fischer & Manstead, 2016; Smith & Mackie, 2016). Likewise, the SEAD model posits that mature empathetic responses are built upon foundational emotional intelligence abilities that are requisite skills for higher-level mastery (Mayer et al., 2016). This conceptualization is vital to the understanding of social emotional ability because without the requisite lower competencies, mastery of sympathetic and empathetic responses will be inhibited. In other words, while an individual may have some capacity to empathize, that individual will not progress towards mastery of the ability without first developing lower-level abilities (Gottman, 1999; Gottman & Declaire, 1997; Gottman et al., 1997; Sue & Sue, 1999).

For example, a parent who has mastered sympathetic and empathetic responses may use empathetic phrases like, “I can tell that hurts,” “What you have told me sounds really sad,” and “I’m really sorry you had to experience this” as they side-step potential power and control issues created by unskilled parental behaviors such as criticism, threats, arguing, verbal or physical force, despair, pleading, and helplessness. Using open communication questions such as “What do you think the solution to this issue is?” or, “This is a problem—how do you think you are going to handle this?” allows a child to own the problem behavior and think about possible solutions while allowing the parent to mentor them in this process of prosocial decision-making and related behaviors (Fay & Cline, 1993; Harris et al., 2015a, 2015b, 2015c; Latham, 1999). Because mastery of social emotional abilities, competencies, and behaviors is highly contextual, the SEAD model allows for this contextualization across and within cultures (Fig. 1).

### Assumptions of the SEAD model

The SEAD model assumes that social emotional abilities vary along a continuum of measurable proficiency and result from interactions between intellectual capacity (afforded by social and emotional intelligences) and contextual circumstances, such as social learning (Mayer et al., 2004; Vygotsky, 1978a, 1978b). Additional assumptions include: (a) Development of higher competency levels of social emotional ability is somewhat linear, but also contextual, and results from bi-directional interactions between the intellectual and developmental critical thinking capacities of social and emotional intelligences and contextual circumstances, such as social learning, as reflected by the arrows or flashes in the SEAD model (Vygotsky, 1978a, 1978b); (b) In a manner similar to Maslow’s *Hierarchy of Needs* (Huitt, 2007), dimensions of the SEAD model are hierarchal and interdependent, again as reflected by the arrows or flashes, in that changes in any specific ability can affect proficiency in other dimensions; and (c) changes in any one specific ability may generalize and bidirectionally influence another social emotional ability proficiency whether in a higher or a lower dimension. Again, the arrows or flashes reflect the potential of these bidirectional influences across and within dimensions and across and within specific abilities, competency levels, and behaviors.

In summary, the arrows in the SEAD model generally reflect the assumption that social emotional ability proficiency develops sequentially in a hierarchical order of sorts both across and within dimensions, with increases in abilities, competencies, and behaviors at lower order levels of the hierarchy both directly and indirectly impacting increases in abilities, competencies, and behaviors at higher order levels of the hierarchy. Development of trait sympathy begins during infancy (Kienbaum, 2014), for example, yet higher order competency and behavior levels of sympathetic abilities develop much later during early adulthood when other related critical thinking and other socio-emotional abilities have matured (Eisenberg et al., 2005; Piaget & Cook, 1952).

A pyramid is intentionally used in the SEAD model to suggest this hierarchy using arrows to indicate bidirectional influences among abilities, competency levels, and behaviors. The SEAD model assumes that abilities to identify and label emotions are foundational to (1) learning to listen to emotions and (2) trusting what they are signaling or indicating. For example, it would be difficult to adequately attend to or interpret emotions if a person were unsure or unable to identify what was personally being felt or what others were feeling.

Correspondingly, as higher competency levels at identifying emotions are achieved, thus increasing individual abilities to attend to, interpret, and regulate emotions and show sympathetic and empathetic response, downward scaffolding may also occur which can be generalized into increased abilities to effectively identify other emotions that were previously difficult or challenging to identify. For example, becoming skilled at identifying, understanding, and accepting the emotion of joy, as well as social and emotional decision-making processes that promote feelings of joy, may result in higher order sympathetic and empathetic response behaviors of showing kindness to others which in turn increase intrapersonal and interpersonal life satisfaction and well-being. The arrows or flashes (Fig. 1) signify that the experienced outcomes from these response behaviors may then be downwardly scaffolded to better identify and understand how anger, a secondary emotion, can inhibit joy as a feeling that leads to life satisfaction and well-being. Recognition of anger as an unpleasant emotion compared to joy may result in motivation to (1) become more skilled at identifying anger early, understanding that it is a secondary emotion usually preceded by a trigger of hurt, pain, or a blocked goal, (2) accepting anger as a valid emotion, and (3) then regulating this anger so it seldom inhibits experiencing the emotion of joy and subsequent life satisfaction and well-being in the future.

Finally, as well as in summary, while a pyramid (rather than concentric conceptualization) best depicts the hierarchical and bidirectional development and progression of the social emotional abilities, competencies, and behaviors discussed heretofore in this treatise, the SEAD model does not assume absolute linearity in this development and progression, due to individual differences in genetic, relational, and sociocultural contexts (Fig. 1; see “Contexts” below; see also Nook et al., 2018). Rather, as noted above, the SEAD model pyramid was intentionally chosen by the authors as the best depiction of how lower order social emotional abilities represent the building blocks foundational to the attainment of higher order competency levels of social emotional abilities and behaviors. Such a hierarchical conceptualization also allows for, for example, the development of social emotional abilities to be bidirectionally scaffolded from a higher dimension (i.e., emotional integration) for one specific ability to a lower dimension (emotional clarity) for another specific ability that may have previously been considered a weakness or deficiency. Additional reasoning for choosing a hierarchical conceptualization for the SEAD model will be discussed in greater detail in a future treatise.

### Emotional theoretical foundations of the SEAD model

According to Scarantino (2016), most emotion theorists concur with the following:

1. Emotion episodes involve, at least in prototypical cases, a set of expressive, behavioral, physiological, and phenomenological features diagnostic of emotions; (2) each diagnostic feature has a range of variability; (3) evolutionary explanations can be given for at least some emotions and/or their components; (4) most aspects of emotions are affected by sociocultural factors; (5) the physical seat of emotions is the brain; (6) emotions motivate actions in distinctive ways; (7) emotions are generally object-directed; (8) emotions have a cognitive basis, consisting of other mental states they presuppose (e.g., memories, perceptions, etc.); (9) emotions can be appropriate or inappropriate with respect to their objects; (10) there are different forms of appropriateness for emotions (e.g., epistemic, moral, prudential); (11) appraisals can help differentiate emotions; (12) appraisals range from primitive to sophisticated forms of information processing; (13) at least some emotions are present in infants and animals; (14) emotions can be in tension with our reflective judgments; and (15) emotions play a functional role in a variety of domains (e.g., rational deliberation, morality, aesthetics). (pp. 36–37)

These theoretical underpinnings of emotion place particular emphasis on sociocultural learning as it affects “most aspects of emotions.” Sociocultural, genetic, and relational contexts which influence SEAD are discussed below.

### Genetic, relational, and sociocultural contextual foundations of the SEAD model

The SEAD model represents a practical approach to the development of social emotional ability. Considerations of context are critical to this practical approach (Fig. 1). Context is defined in this treatise as the circumstances or events in which something exists or takes place (Merriam-Webster, 2021). Genetic, relational, and sociocultural contextual foundations of the SEAD model include the following:

- *Genetic contexts* which shape the individual development of social emotional ability include all heritable influences, such as physiology, neurology, and temperament (e.g., habituation, neuroplasticity, neuropeptide protein transmission);
- *Relational contexts* which influence SEAD include all intrapersonal and interpersonal interactions (e.g., individual, spouse, family, children, relatives, peers, role models, and other antagonists and protagonists);
- *Sociocultural contexts* which shape SEAD include all social, political, economic, cultural, educational, and historical influences, such as cultural norms, group membership, social position, laws, work, media, neighborhood, school, historic events—(e.g., 9/11, COVID-19).

### Vygotsky’s sociocultural theoretical foundations of the SEAD model

Vygotsky’s sociocultural theory of development (SCTD) (1978a; 1978b) provides clear, parsimonious, and logical constructs to explain the contextual processes and influences of complex cognitive, emotional, and social development (John-Steiner & Mahn, 2012). While the SEAD model is not intended to address the specific genetic or neurological contextual processes of childhood social emotional development, Vygotsky’s sociocultural theory can describe how social emotional abilities are relationally experienced and influenced by contextual interactions between cognitive capacity and environmental circumstances through social learning over time.

The major assumptions of sociocultural theory mirror those of the SEAD model and include: (a) contextual learning results from interaction between cultural and social environments; (b) contextual learning within these environments is dependent upon the presence of specific inherited (Nikolova et al., 2016) and learned cognitive abilities, and is typically guided by instruction from a more knowledgeable other; and, (c) language (e.g., symbols representing emotions) constructs and transforms relational and sociocultural contextual development through interactive guided participation (Fani & Ghaemi, 2011; Kraker, 2000; Shabani, 2016). The major concepts of the sociocultural theory of development (SCTD) align with the SEAD model, describing the hierarchal nature of increasingly complex learning represented by the SCTD through the: (a) level of current contextual ability; (b) zone of proximal development; and (c) scaffolding (Fani & Ghaemi, 2011; Kraker, 2000; Mishra, 2013; Shabani, 2016).

#### Level of current contextual ability

Level of current contextual ability is defined in the current treatise as the competency level of an individual’s cognitive, social, and emotional ability achieved at given points in time across the lifespan. This represents the dynamic area of knowledge and ability that expands as individuals learn and grow through socialization in their given contextual environments (Fine & Fincham, 2013; Lewis, 2016; Mather & Ponzio, 2016; Sommerville, 2016). Language (verbal and non-verbal) acts as both a sign and as a tool to guide this socialization process, usually spearheaded by the influence of at least one knowledgeable other (Daniels et al., 2007; John-Steiner, 2007). Cultural norms, group membership, social position, developmental age and stage, comfort with familiar situations and fear of novel ones, and dyadic interactions with siblings, peers, and partners are good examples of how and when language influences levels of current contextual social emotional ability (Daniels et al., 2007; Vygotsky, 1978a, 1978b, 1981).

Indeed, sociocultural theory focuses on the multiple layers of the social environment (e.g., family, community) that interact with one another and the influence these environments have on individual adaptive and maladaptive abilities to fulfill their own and others’ needs. Because of the immediate and repeated influence of the family, for example, family members typically represent the primary contextual socializer of current and intergenerational thought, emotion, and behavior, and thus how strategies for meeting individual and collective needs are perceived, felt, and enacted (Fig. 1).

#### Zone of proximal development

The zone of proximal development is defined as a collection of tasks dependent upon an individual’s inability to complete the tasks without the assistance of a teacher, mentor, or guide. Specifically, tasks that are just outside the grasp of one’s level of current abilities, but can be accomplished with the support, instruction, guidance, and modeling of more knowledgeable others such as parents, siblings, peers, teachers, and counselors are said to be in one’s zone of proximal development (Fine & Fincham, 2013; Kraker, 2000; Vygotsky, 1981; Widen, 2016).

#### Scaffolding

**Fig. 2:**
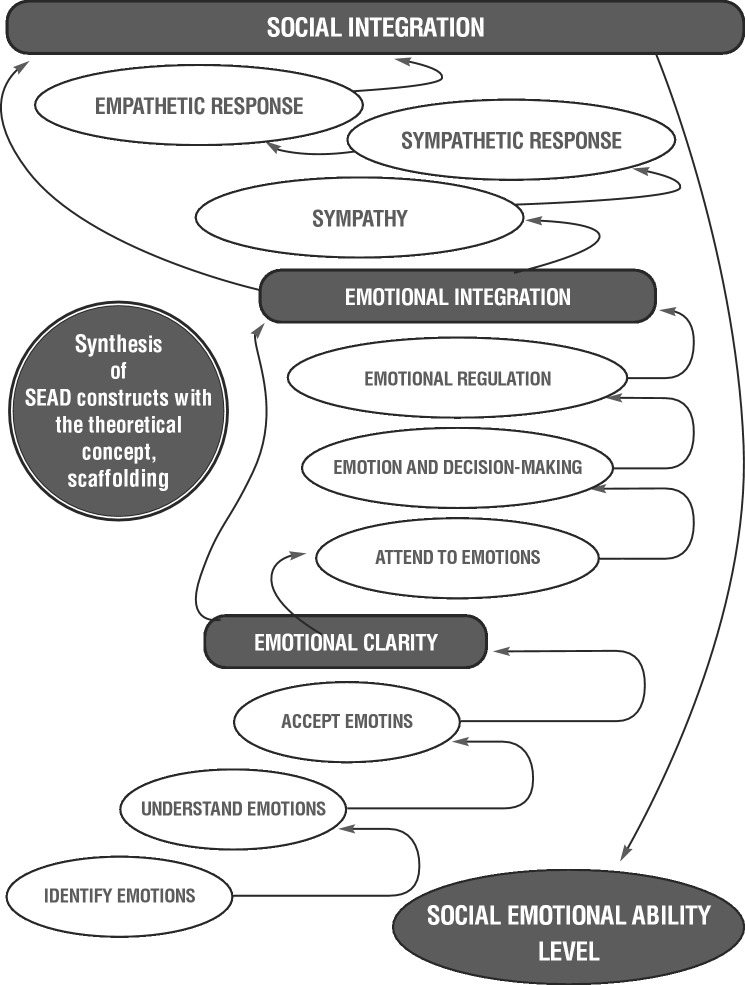
Synthesis of the SEAD and the sociocultural theory of development

Scaffolding is defined as the process by which lessons are provided through careful and incremental guidance from more knowledgeable others to support the learning of more complex tasks (Fine & Fincham, 2013; Mishra, 2013; Vygotsky, 1981). As previously noted, this treatise explores processes of emotionally supported and scaffolded abilities critical to effective social interaction, as differentiated by the progression of nine discrete social emotional abilities, and the ways in which these abilities improve over time. As a theory concerned with learning and development across the lifespan, the SCTD provides a framework for understanding the relationship between the constructs of the SEAD model because these constructs are dependent upon the development of increasingly complex applied skills through interactions between intellectual capacity, environmental circumstances, and social learning. Figure 2 illustrates the integration of the SEAD constructs with the bidirectional nature of scaffolding. A majority of the scaffolding occurs hierarchically or upwardly with the caveat that some scaffolding can occur vertically as well as nonhierarchically or downwardly, as discussed previously.

Verenikina (2010) posited that the sociocultural perspective appropriately explains the processes of learning and developing increasingly complex skills. This perspective views learning as a progression from elementary mental functioning to higher order and more complex abstract mental functioning facilitated through interactions between emotional and cognitive capacity and thousands of social experiences (Fischer & Manstead, 2016; John-Steiner & Mahn, 2012). Huitt and Dawson (2011) articulated that the SCTD explains social, emotional, and cognitive skill development as a process in which individuals expand their social emotional abilities beyond existing levels of competence through imitation and instruction which facilitates the completion of tasks within their zone of proximal development. As tasks are completed, learning occurs and is internalized, typically resulting in adaptive development. Internalization is defined as the process of incorporating attitudes or behavior into one's nature through learning, assimilation, or accommodation (Fine & Fincham, 2013; Piaget & Cook, 1952).

A framework of complex scaffolding generally facilitates the development of progressively more complex cognitive, social, and emotional skills (Fine & Fincham, 2013; Verenikina, 2010; Vygotsky, 1981). As noted above, scaffolding can also occur bidirectionally wherein the achievement of specific higher order abilities can then be generalized and scaffolded vertically or downwardly to improve social emotional abilities in lower order social emotional abilities previously considered weaknesses. Thus, the development of supportive social interaction holds important implications for the expansion of social and emotional skills, as new knowledge is created and generalized when individuals internalize social learning appropriated through social participation (Vygotsky, 1978a, 1978b). The development of social emotional ability is rooted in these processes, and in the subsequent interactions between cognitive capacity and contextual factors.

## Situating the SEAD model within human development, social, and family theories

In addition to emotion and sociocultural theory, the SEAD model is consistent with the assumptions and perspectives of other human development, social, and family theoretical perspectives as cursorily outlined below.

### The SEAD model and human development theory

The authors assert that the SEAD model is consistent with current human development theory and research for at least the following six reasons:

1. The SEAD model assumes that the development of social emotional ability is continuous, gradual, and predictable according to age and stage of development (Grossman, 2018; Grossman et al., 2018; Lewis, 2016; Shaver et al., 2016; Wilson-Mendenhall & Barsalou, 2016).
2. The SEAD model assumes that social emotional ability is generally sequential and hierarchical in that the development of lower order abilities are typically followed by the development of specialized higher order abilities (Adolphs & Anderson, 2018; Barrett, 2018; Tomasello & Vaish, 2013).
3. The SEAD model assumes that social emotional abilities can also be bidirectional in their influences in that higher order abilities in one specific ability area and dimension can also be scaffolded and positively influence lower order abilities in another specific ability area and dimension (Harris et al., 2016; McKown et al., 2009; Rojas & Abenavoli, 2021).
4. The SEAD model assumes because social emotional ability is both sequential and hierarchical that sensitive and critical periods may influence the varying rates of cognitive capacity, growth and development from individual to individual (Feldman, 2015; Gee, 2016; Heckman, 2014; Nelson & Gabard-Durnam, 2020; Woodard & Pollak, 2020).
5. The SEAD model assumes that social emotional cognitive capacity, growth, and development are a result of a unique, ongoing combination of genetic, relational, sociocultural and individual influences and choices, that they are constantly interacting, and that they influence all aspects of the development of social emotional ability (Malik & Marwaha, 2020; McKown et al., 2009; Stern & Cassidy, 2018; Stern et al., 2021; Tottenham, 2017).
6. The SEAD model assumes that SEAD is functional in that it can lead to positive changes and increases in life satisfaction and well-being (Fredrickson, 2016; Kuppens et al., 2008; Sánchez-Álvarez et al., 2016)

### The SEAD model and social and family theory

The authors assert that the SEAD model is consistent with current social and family theory for the following seven reasons:

1. The SEAD model assumes that social emotional learning occurs at all levels of social and relational development (Hoffman, 2009; Lane, 2000; Napolitano et al., 2021; Weissberg et al., 2015; White et al., 2014; Wilson-Mendenhall & Barsalou, 2016).
2. The SEAD model assumes that adaptive intrapersonal and interpersonal social emotional ability change is continuous throughout the creation, maintenance, and dissolution of the relationship development lifecycle (Brackett et al., 2015; Clore & Schiller, 2016; Mather & Ponzio, 2016; Nichols & Davis, 2020; White et al., 2014).
3. The SEAD model assumes that social emotional ability symbols are given different meanings and emphasis across cultures and that these meanings and emphasis influence social emotional intrapersonal and interpersonal comprehension, interaction, and worldviews (e.g., linear, relational, individualistic, collectivist) (Cross, 1997; Hoemann, et al., 2019; Koltko-Rivera, 2004; LaRossa & Reitzes, 2009; Linask, 2019; Lindquist et al., 2016; Sue & Sue, 1999).
4. The SEAD model assumes that social emotional learning is contextual in that individuals and groups are socialized in the development of both general and specific social emotional abilities similarly and differently across cultures (Mesquita et al., 2016; Sue & Sue, 1999; Vygotsky, 1978a, 1978b, 1981; White et al., 2014).
5. The SEAD model assumes that both general societal systems (e.g., chronosystem, macrosystem, exosystem, mesosystem, microsystem) and more specific family systems (attachment, boundaries, sub-systems, socialization, feedback) influence individual, within group, and between group social emotional ability development (Bronfenbrenner, 1979; Bubolz & Sontag, 2009; Whitchurch & Constantine, 2009; White et al., 2014).
6. The SEAD model assumes that conflict is inevitable in intrapersonal and interpersonal interaction and that power, resource, opportunity, and other disparities influence changes in social emotional abilities (Farrington & Chertok, 2009; Van Kleef & Chang, 2020; Vaish et al., 2008; White et al., 2014).
7. The SEAD model assumes that maximizing benefits and minimizing costs which result in increases to life satisfaction and well-being are a primary motivation in the development of social emotional abilities (Lerner et al., 2015; Sabatelli & Shehan, 2009; White et al., 2014).

## Justification for constructs of the SEAD model

The background information cited above represents the underlying framework for justification of the specific constructs of the SEAD model discussed below.

### Emotional clarity

Emotional clarity is the first construct of the SEAD model and represents the initial integration of emotion and thought into a knowledge-based skill. Emotional clarity is defined as the ability to identify, understand, and accept one’s emotional experiences (Boden et al., 2013; Flynn & Rudolph, 2010; Shallcross et al., 2010). Emotional clarity was abstracted from self-awareness (Flynn & Rudolph, 2014), a construct of emotional intelligence theory. Goleman (2004) asserted that self-awareness provides the foundation upon which emotional intelligence theory is based. He further defined dimensions pertinent to emotional intelligence as the abilities to recognize, understand, and view one’s own emotions without criticism (acceptance). Emotional clarity also supports later development of the ability to recognize and respond thoughtfully to emotional events in others.

Building knowledge is a process that begins with elementary mental functioning and progresses with increasingly more complex mental functioning through scaffolding (Mishra, 2013). The individual dimensions of emotional clarity represent the progression from the less complex foundational ability to identify, to the slightly more complex ability to understand, to the more complex ability to accept one’s emotions (Harris et al., 2016; Lewis, 2016; Piaget & Cook, 1952). Mishra (2013) asserted that this progression from less complex to more complex parallels concepts of the sociocultural theory of development (Vygotsky, 1978a, 1978b, 1981).

The ability to identify one’s emotions is a basic cognitive function necessary for understanding them (Gottman et al., 1997). Understanding, or comprehending one’s emotions is a more complex function than the ability to identify them and is analogous to trying to learn to solve a math problem without first learning to count (Harris et al., 2016). Therefore, the level of development of one’s ability to understand emotions would partially depend upon the level of one’s ability to identify emotions.

Similarly, the cognitive ability to accept and embrace emotions, particularly negative emotions, is comparatively more abstract and slightly more complex than the abilities to identify and understand emotions (Friel & Friel, 1998; Wilson-Mendenhall & Barsalou, 2016). Thus, the SEAD model posits that mastery of emotional clarity can best be explained as a product of scaffolding, as individual dimensions of intellectual capacity provided by emotional intelligence interact with genetic, relational, and sociocultural contextual factors to provide the increasingly complex development of the abilities to identify, understand, and accept one’s emotions.

It should be noted that emotional clarity represents foundational knowledge that, once mastered, remains relatively static or routine (Wilson-Mendenhall & Barsalou, 2016). Even though the SEAD model is primarily concerned with socially adaptive intellectual and behavioral responses, emotional clarity is necessarily the foundational construct of the SEAD model, as emotional clarity supports subsequent development of important emotional skills, such as the ability to understand emotional displays in others and emotional regulation capabilities in oneself (Fischer & Manstead, 2016; Flynn & Rudolph, 2010, 2014; Gratz & Roemer, 2004). Emotional clarity is related to the development of more sophisticated social emotional abilities; those with higher levels of emotional clarity develop higher levels of the ability to regulate their emotions (Fischer & Manstead, 2016; Flynn & Rudolph, 2010; Nolen-Hoeksema, 2012; Suri & Gross, 2016). Higher levels of emotional clarity are also positively related to subjective well-being and adaptive attributional styles (Boden et al., 2013; Flynn & Rudolph, 2010). Conversely, people with lower levels of the ability to understand their emotions expend greater effort managing emotions and have more difficulty with goal-oriented behaviors (Flynn & Rudolph, 2010). Furthermore, lower levels of emotional clarity are associated with contextually inappropriate and less adaptive stress responses (Flynn & Rudolph, 2010; Ganzel et al., 2016).

#### Identifying emotions

The ability to identify emotions is the first of three dimensions of the emotional clarity construct. The ability to identify emotions underpins development of all social emotional abilities and is defined as the ability to recognize, name, and label emotions, as well as differentiate emotional states (Boden et al., 2013; Goleman, 2004; Gottman et al., 1997). Identifying emotions is a learned process begun in infancy, when perceptions of emotional signals are undifferentiated (Lewis, 2016; Widen, 2016). The ability to differentiate and identify emotions normatively increases with developmentally appropriate language (Lindquist et al., 2016) and experiences (Kujawa et al., 2014; Widen, 2016). However, differentiated emotional expressions can be quite subtle (Wells et al., 2016; Wilson-Mendenhall & Barslou, 2016), and lower levels of emotional processing ability can have adverse effects on social decision-making (Fischer & Manstead, 2016; Kujawa et al., 2014).

#### Understanding emotions

The ability to understand emotions is the second dimension of the construct, emotional clarity, and is defined as the ability to comprehend the meaning of emotions and to know their nature and intensity (Helm, 2009; Mayer et al., 2012). The ability to understand emotions would not be possible without first having the ability to identify emotions. This ability begins to develop in childhood (Lewis, 2016; Nichols et al., 2010; Widen, 2016). Children who are given clear emotional messages generally develop a greater understanding of the nature of emotional meanings and expressions than children who do not (Gottman et al., 1997; Parker et al., 2013). For example, infants react to changes in their mothers’ emotional expressions according to intensity level cues and their own understanding of the intended emotion (Bar-On & Parker, 2000; Walker-Andrews et al., 2011).

Development of the ability to understand emotions has important implications for children as they grow and mature into adulthood because the ability to understand emotions and their intensity is essential for emotional health (Bar-On & Parker, 2000; Kubzansky & Winning, 2016; Parker et al., 2013). Children who have difficulty understanding emotions are at risk for poor social interactions and impeded friendship formation (Parker et al., 2013; Spackman et al., 2006). Understanding emotions requires specific cognitive appraisal abilities to interpret emotions (Brackett & Salovey, 2006; Brackett et al., 2016), particularly within diverse cultural contexts. Differences in cognitive appraisals may include perceptions of how emotions are experienced within relational and linear, individual and collectivist, and religious and secular contexts and how specific emotions are perceived to either promote or violate moral norms (Cross, 1997; Limb & Hodge, 2008; Mesquita et al., 2016).

#### Accepting emotions

The ability to accept emotions is the third dimension of the construct, emotional clarity. The ability to accept emotions is the capstone of the integration of emotion and thought into a knowledge-based skill and is critical to the subsequent development of emotional regulation (Fischer & Manstead, 2016), a fundamental component of emotional intelligence (Mayer et al., 2016; Shallcross et al., 2010). Emotional acceptance is defined as the cognitive ability to accept and embrace emotions, as opposed to the denial or avoidance of emotional experiences. The ability to skillfully accept emotions is contingent upon the ability to both identify and understand emotions. Gratz et al. (2007) posited that the acceptance or avoidance of one’s emotions is rooted in experiences encountered in childhood, specific to attachment experiences (Bowlby, 1979; Cassidy, 1999).

The ability to accept one’s emotions, particularly the acceptance of negative emotions (e.g., fear, anger, sadness), is healthy, useful, and adaptive; conversely, non-acceptance of emotions often results in unhealthy emotional processes (Gottman et al., 1997; Shallcross et al. 2010). Furthermore, non-acceptance may result in secondary emotions being substituted for primary emotions in order to avoid experiencing perceived uncomfortable emotions (Ekman, 2007; Greenberg, 2004). For example, as noted previously, anger is considered a secondary emotion often substituted for emotions such as fear, anguish, jealousy, frustration, and perceptions of blocked goals (Friel & Friel, 1998; Greenberg, 2004; Hendricks et al., 2013). Additionally, individuals may experience emotional stimuli such as hurt, anger, jealousy, or desire, and deny these feelings and consequently respond reflexively in counterproductive ways, such as through the expression of lust (Ekman, 2007; Friel & Friel, 1998; Hendricks et al., 2013). The ability to recognize and accept the full range of perceived emotions is socially useful and is an important component of social emotional ability (Gottman et al., 1997; Momm et al., 2015).

### Emotional integration

Emotional integration is the second and central construct of the SEAD model. According to Gu et al. (2013), all complex human behavior is determined by the integration of emotional and cognitive processes. Emotional integration is defined as the ability to employ aspects of one’s emotions such as motivation, inspiration, and creativity to assist in decision-making and the deployment of contextually adaptive emotional regulation behaviors. The dimensions of emotional integration are abstracted from emotional intelligence theory (Mayer et al., 2016). Emotional integration marks the initial shift from intrinsic cognitive processes of emotional clarity toward the integration of emotion with cognition and extrinsically focused responses. Specifically, emotional integration marks the point at which proficiency in social emotional ability begins to manifest intellectual and behavioral responses beyond heritable intellectual capacity through interaction with relational and sociocultural environmental circumstances.

The development of emotional integration is dependent upon the development of emotional clarity (Boden & Thompson, 2017; Thompson et al., 2017). The sequencing of consciously attending to one’s emotions and purposefully incorporating emotions into the decision-making and behavioral selection processes represents progressively more complex scaffolding in support of the development of the highly complex and abstract social emotional ability to manage emotions appropriately (Fine & Fincham, 2013; Goleman, 1995; Hajcak et al., 2016; Mishra, 2013). Progressive integration of emotion and thought and adaptive behaviors parallels the development of higher mental processes described by concepts of the sociocultural theory of development (SCTD) and theory of social emotional ability development (SEAD).

Clearly, integrating emotion and thought into the decision-making process to assist in the deployment of contextually appropriate behaviors is a more complex and abstract function than the ability to integrate emotion with thought to assist in emotional interpretation (Lempert & Phelps, 2016; Wilson-Mendenhall & Barslou, 2016). Furthermore, the ability to respond to and manage emotions is comparatively more complex and abstract than the abilities to pay attention to, understand, and accept emotions. Thus, the construct of emotional integration can best be explained by scaffolding and individual levels of development of emotional integration abilities that are dependent upon the levels of development of progressively more complex abilities to interpret, respond to, and regulate emotions (Fischer & Manstead, 2016).

#### Attending to emotions

The ability to attend to emotions is the first of three dimensions of the construct of “emotional integration,” and is fundamental to emotional intelligence (Mayer et al., 2016). This ability is defined as the extent to which individuals and groups value and focus cognitive attention on (i.e., apply initial reasoning to) their emotions (Hajcak et al., 2016; Smith & Mackie, 2016). More specifically, while basic attentional processes typically precede conscious identification or understanding of emotion and can even occur below the level of conscious awareness, attending to emotions as defined here refers to the ability to consciously focus attention on interpreting, or “listening” to what an emotion is indicating. Applying attentional interpreting and initial reasoning to emotions is important to the quality of social engagement because emotional signals generally contain important messages about interactions with others (Lane, 2000; Richter, 2015; Schwarz, 2000).

Attending to emotions is not included in the foundational construct, emotional clarity, because it is conceptualized in the SEAD model as a higher-ordered attentional ability more closely associated with reasoning processes necessary to the development and maintenance of emotional processing, decision-making, and regulation (Fischer & Manstead, 2016; Mishra, 2013). In other words, it is possible to have a basic attentional ability to identify, understand, and accept emotions and yet lack the higher-order attentional ability to adequately interpret emotions, determine emotional meaning, integrate that meaning into behavioral decision-making, and regulate emotions once emotional meaning is processed and conceptualized.

#### Emotion and decision-making

The ability to integrate emotion into decision-making is the second of three dimensions of the construct, emotional integration, and is fundamental to emotional intelligence (Mayer et al., 2016). Integrating emotion into decision-making is the intentional incorporation of emotional meaning into cognitive decision-making processes which support choosing contextually appropriate behaviors (Clore & Schiller, 2016; Greenberg, 2016; Lindquist et al., 2016).

Decision-making is a cognitive process that relies on emotional signals (Schwarz, 2000); therefore, the ability to accurately interpret emotional meaning supports development of the ability to adaptively respond to emotions (Fischer & Manstead, 2016). Individuals with higher competency levels of social emotional ability tend to “listen” to their emotions and are more likely to trust what they indicate. They are therefore better able to use emotions to facilitate thoughtful decision-making and implement contextually appropriate behavioral responses (Ekman, 2007; Lerner et al., 2015; Mayer, Roberts, et al., 2008; Mayer, Salovey, et al., 2008). Accurate emotional interpretation supports healthy decision-making and contextually adaptive behavioral response. However, it is possible to identify emotions but not to accept them, thereby limiting the ability to interpret them accurately and respond appropriately (Friel & Friel, 1998; Greenberg, 2016; Kring & Mote, 2016). Decision-making without an accurate interpretation of emotions can lead to profoundly negative social outcomes (Bar-On & Parker, 2000; Kring & Mote, 2016; Zheng et al., 2017).

#### Emotional regulation

The ability to regulate emotions is the third dimension and is vital to the construct, emotional integration. Emotional regulation is a fundamental component of emotional intelligence (Mayer et al., 2016), and is defined as the ability to modulate emotional experiences intentionally and consciously (Chapman et al., 2011; Fischer & Manstead, 2016). Development of the ability to manage emotions is primarily facilitated through the ability to respond thoughtfully to emotions (Wilson-Mendenhall & Barslou, 2016). Components of emotional regulation include managing distress, controlling emotional expression, setting appropriate priorities, and sustaining motivation (Broderick & Jennings, 2012; Sands et al., 2016).

Emotional regulation is widely reported as critical to prosocial human development and behavior. An abundance of evidence is available regarding the impact of emotional regulation on social engagement (Cohen, 2012; Ivcevic & Brackett, 2014; Lopes et al., 2005, 2011; Silk et al., 2003). Two primary methods are used to regulate and manage emotions: reappraisal and suppression. Those who reframe, reinterpret, and respond to emotions in adaptive ways rather than suppress their emotions in maladaptive ways are more likely to experience positive emotions, sociality in their relationships, and subsequent well-being (Gross & John, 2003; Zheng et al., 2017). According to Ochsner et al. (2012), explicit emotional regulation strategies are deployed through cognitive selection from an array of behavioral choices. Emotional regulation begins in childhood (Denham et al., 2012; Gottman et al., 1997) and the ability to manage emotions develops across the lifespan (Allen & Nelson, 2018; Fischer & Manstead, 2016; Sands, et al., 2016; Suri & Gross, 2016).

According to Silk et al. (2003), emotional regulation can increase mental health, improve personal relationships, and reduce the risk for psychopathology. Furthermore, those with higher levels of emotional regulation skills often view themselves as socially aware and prosocial (Lopes et al., 2005). Additionally, the management of negative emotions is positively associated with one’s ability to understand emotions (Denham et al., 2010).

Emotional dysregulation is responsible for a wide range of social, emotional, and behavioral problems (Broderick & Jennings, 2012; Greenberg, 2016; Kring & Mote, 2016). Emotional dysregulation has been shown to reduce social interactions and promote aggressive coping styles, which have been shown to prolong and heighten conflict (Wilton et al., 2000).

### Social integration

Social integration is the SEAD model’s third and highest ordered construct. Social integration is defined as the integration of one’s emotions, thoughts, and behaviors with adaptive sympathetic and empathetic social interactions with others. The dimensions that define the construct of social integration are foundational to social intelligence (Rahim, 2014; Srivastava & Das, 2016). Sympathetic response tends to facilitate immediate social cooperation and empathetic interaction tends to facilitate improved long-term social engagement; both sympathetic and empathetic responses form the basis for most social interaction and prosocial behavior (Decety & Michalska, 2010; Irwin et al., 2008; Keltner, et al., 2016; Zaki & Ochsner, 2016). Improved social engagement is an important predictor of well-being, life satisfaction, and happiness (Baumeister et al., 2013; Lambert et al., 2010; Rohrer et al., 2018).

The development of social integration is a highly complex and abstract social emotional ability that is supported by emotional clarity and emotional integration. Social integration results from increasingly complex scaffolding that helps one develop the ability to comprehend the emotional states of others and thereby interact with others through sympathetic and empathetic prosocial behaviors. Decety and Michalska (2010) asserted that a progressive relationship exists between sympathetic response and empathetic response that parallels the development of higher mental processes described by concepts of the SCTD, as sympathy is strongly related to effortful control, and children with greater effortful control display greater empathic concern. Developing the ability to respond empathetically to others, which is more complex and abstract than the ability to respond sympathetically, is at least partially dependent on the ability to respond sympathetically (Eisenberg, 2000; Eisenberg et al., 2001, 2005; Spinrad & Eisenberg, 2017). Thus, the summary construct of social integration can best be explained by scaffolding where individual levels of social integration abilities are dependent upon the development of progressively more complex abilities to respond to the emotions of others, both sympathetically and empathetically.

#### Sympathy and sympathetic response

The ability to respond sympathetically to others is the first of three dimensions of the social integration construct. Sympathy is defined as a concern resulting from comprehension of the emotional distress of others, accompanied by a desire to alleviate that distress (Decety & Michalska, 2010; Eisenberg, 2000; Jeffrey, 2016; Spinrad & Eisenberg, 2017). The development of sympathy begins in early childhood and is associated with the secure or insecure nature of the child’s attachment system (Bowlby, 1979; Cassidy, 1999). A mother’s support can serve as a protective factor in the development of sympathy by buffering children against unsupportive relationships (Kienbaum, 2014; Laible & Carlo, 2004). Children normatively begin development of sympathetic responses from primary caretakers through imitative learning. Additionally, adolescents who score high in trait sympathy also score high in moral judgment, which is known to motivate prosocial behavior (Bowlby, 1979; Eisenberg et al., 2001; Malti et al., 2012).

The definition of sympathy also includes the concern or apprehension that may be felt by a sympathizer as a consequence of personal boundaries being violated by others who may be emotionally distressed or disturbed (Eisenberg, 2000; Spinrad & Eisenberg, 2017). Personal concerns associated with sympathetic comprehension and apprehension are integral to understanding sympathetic response. The ability to respond sympathetically is a complex, abstract, high mental function necessary to the development of higher levels of social emotional ability. The development of the ability to respond sympathetically relies upon the preceding social emotional abilities discussed above, with the abilities to regulate emotions, and to recognize, understand, and accept the emotional states of others as being particularly relevant. Responding sympathetically represents the initial interpersonal integration of emotion, cognition, and behavior with others through social emotional interaction.

#### Empathy and empathetic response

Empathy is the second dimension of the construct, social integration, and empathetic response is the third. Empathy is defined as the ability to experience what others are feeling, including the comprehension and vicarious experience of the emotional states of others (Eisenberg, 2000; Lockwood, 2016; Paulus et al., 2013; Zaki & Ochsner, 2016).

Likewise, empathetic response is defined as the ability to adaptively participate empathetically through social interaction. Empathetic response is the capstone of the SEAD model. It plays a central role in social interaction and can be conceptualized as interactions wherein one person vicariously shares and experiences the feelings of another person (Decety & Michalska, 2010) and intentionally attempts to increase this person’s well-being. Zaki and Ochsner (2016) identified the empath as a *perceiver* and the “other” as the *target* and reported that experiencing empathy does not always increase the well-being of the perceiver (e.g., empathy fatigue) and empathic response does not always increase the well-being of the target (e.g., psychopathology). Higher levels of empathy are generally, however, negatively related to conflict and positively related to prosocial problem-solving behaviors; specifically, people who are more emotionally responsive to others when faced with conflict may inhibit antisocial responses (Wied et al., 2007).

The abilities associated with emotional clarity and emotional integration, and particularly the social emotional ability to adaptively respond sympathetically to others, support development of the scaffolded ability to adaptively respond to others empathetically. Empathetic response is considered the highest order of social emotional ability because it generally unifies each of the other components of the SEAD model into a holistic and comprehensive ability to negotiate social emotional states and contexts effectively (with some noted exceptions, such as psychopathology and empathy fatigue); therefore, it can be thought of as the capstone of SEAD theoretical model.

#### Situating sympathy, empathy, and prosocial behaviors within the SEAD model

In this section, the authors address the following two questions in an attempt to further situate and integrate the SEAD model with the constructs of sympathy, empathy, prosocial behaviors and current research: (1) Why sympathy precedes empathy in the hierarchical order of the SEAD theoretical model and not the other way around? (2) What are the potential associations between sympathetic and empathetic response and prosocial behaviors?

In answer to the first question, efforts to distinguish sympathy from empathy theoretically and psychometrically have proven to be challenging (Cuff et al., 2014; Hojat et al., 2011; Wang et al., 2017). Cuff et al.’s (2014) review of literature found 43 distinct definitions of empathy, more than ten of which merged empathy with sympathy. These authors concluded along with Hein and Singer (2008) that put simply, empathy generates observer (i.e., perceiver) emotions associated with *feeling as* another person (i.e., target) might be feeling by vicariously experiencing the *same or similar set* of emotions, such as deep sorrow or sadness. Sympathy generates a *different set* of observer emotions altogether than empathy, such as emotions which are more closely associated with *feeling for* a target, particularly concern (see Singer & Lamm, 2009). Given their review and additional neurological information (Decety & Jackson, 2004), Cuff et al. (2014) concluded that there should be two separate terms, *sympathy* and *empathy*, for distinguishing how observers both *reason* and *feel* about the targets they are observing, the former representing a lower ordered set of reasoning and feeling abilities than the latter, a finding which is consistent with the SEAD model.

Also consistent with the SEAD model are Zaki and Ochsner’s (2016) findings who defined empathy as “the ability and tendency to share and understand others’ internal states” and then discussed empathy in terms of two-related processes: (1) experience sharing “(i.e., ‘the tendency of *perceivers* (individuals focusing on someone else) to take on the sensorimotor, visceral, and affective states of *targets* (individuals on whom perceivers focus); and (2) mentalizing (i.e., perceivers’ explicit reasoning about targets’ internal states using ‘lay theories’ about how situations produce internal states” (pp. 871–872). Similar to Cuff et al. (2014), these authors distinguished the empathetic mentalizing process from the “simpler” sympathetic mentalizing process of “merely accessing information about targets’ states or traits” out of concern, which tends to be more rapid and spontaneous.

Because empathy involves greater breadth and depth of emotional comprehension, mentalizing, and perspective taking than sympathy, it is considered a higher order ability in the SEAD model. Thus, sympathy precedes empathy in the hierarchical order of the SEAD theoretical model and not the other way around. In a future treatise, the authors will parse out and identify additional characteristic nuances between sympathy and empathy as situated within the SEAD model.

In answer to the second question, the authors intentionally differentiated sympathetic response from empathetic response and prosocial behaviors for the following reasons: (1) to clarify important differences between terms; (2) to highlight some individual differences in empathy-related responses to emotional situations; (3) to identify potential differences in speed, spontaneity, and origins of sympathetic and empathetic response behaviors; and (4) to explain the authors’ reasoning for why empathetic response is considered a higher order construct when compared to sympathetic response in the SEAD model.

First, Zaki and Ochsner (2016) described a third aspect of empathy as “*prosocial motivation*, through which individuals who share and understand targets’ states are often compelled to help those targets” (pp. 871–872) through prosocial behaviors. In the SEAD model, empathetic and sympathetic response behaviors are considered synonymous with prosocial behaviors. The term “response behaviors,” however, is specifically used in the SEAD model to identify the purposeful intent of perceivers to respond to the cues and needs of others, which is not necessarily inferred by the use of the term “prosocial behaviors.” Additionally, the terms “sympathetic response” and “empathetic response” are intentionally used to differentiate the prosocial motivations behind observers or perceivers enacting sympathetic and empathetic responses toward their targets (i.e., *feeling for* compared to *feeling as,* respectively) (Cuff et al., 2014; Spinrad & Eisenberg, 2017).

Second, Spinrad and Eisenberg (2017) identified some important individual differences in empathy-related responding (i.e., empathetic, sympathetic, prosocial, personal distress) to emotional situations below. These insights help to explain the diverse array of empathetic and sympathetic responses of perceivers to their targets.

1. Susceptibility to emotional overarousal is negatively associated with sympathy and prosocial behaviors.
2. Prosocial behaviors directed at targets tend to promote internalized positive beliefs in perceivers about themselves and, therefore, the likelihood that prosocial behaviors will continue.
3. Positive emotionality is positively associated with openness to emotional signals and information, social competence, sympathetic and empathetic responsiveness to others’ needs, and the empowered belief that these needs can actually be met.

Third, because sympathy typically involves a perceiver’s positive emotional *concern for* a target’s well-being and the accompanying desire to alleviate suffering, sympathetic prosocial response behaviors often occur more quickly and spontaneously than empathetic prosocial response behaviors (Spinrad & Eisenberg, 2017). Spinrad and Eisenberg (2017) also noted that “sympathy may stem from empathy (but can also be derived directly from perspective taking or other cognitive processes such as retrieving information from memory)” (p. 332), which can subsequently influence the speed and spontaneity of sympathetic response behaviors.

Fourth, differences in the depth and the degree of experience sharing, mentalizing, and perspective taking is the reasoning behind distinguishing empathy and empathetic prosocial response behaviors as higher order constructs when compared to sympathy and sympathetic prosocial response behaviors in the SEAD model (Zaki & Ochsner, 2016).

This distinction may especially be evident when perceivers are confronted with making difficult decisions that require more cognitively complex emotional reasoning than a concern to relieve suffering. While sympathy may be motivated by the concern of a perceiver *for* a target to relieve suffering, in the SEAD model the higher order construct of empathy may require perceiver reasoning abilities “to take on the sensorimotor, visceral, and affective states of *targets*” which can help the perceivers to more fully meet the target “where they are” in ways that are more socially and emotionally meaningful and powerful (Zaki & Ochsner, 2016).

It is one thing, for example, to express concern for a target’s situation and to respond sympathetically to an immediate situation in an attempt to relieve a target’s suffering, and yet quite another to do the hard prosocial work of “being there” for the target in the often difficult and challenging ways necessary to truly help them meet their needs in both the short- and long-term. Caretaking of an ailing family member is a good illustration of how initial sympathetic prosocial response may turn to empathetic prosocial response as the requests and needs of the ailing family member are honored and met as much as possible during the challenging mental, social, emotional, and physical declines that occur over time.

In a future treatise, the nuances between sympathetic and empathetic prosocial response behaviors will be also addressed. A summary of the unique contributions of the SEAD model to the social emotional body of knowledge is described in the next section.

## Summary of unique contributions of the SEAD model

In this treatise the authors explored the need for a practical developmental model abstracted from social intelligence and emotional intelligence theories to define, differentiate, and provide diagnostic-level measurement of salient dimensions specific to social emotional ability development. The authors provided linkages from the research literature to support the argument that individual, family, parent–child, and dyadic couple relationships are fundamental to the development of emotional intelligence and social emotional competence during early childhood, the trajectory of later childhood development in general, and the development of social emotional ability across the lifespan. The authors proposed a new simplified and practical developmental model specific to social emotional ability, the SEAD model, and provided guidance from the literature supporting its component structure.

Linkages were also provided that explain the systematic, hierarchal progression of constructs of the SEAD model. These linkages also demonstrated the generally progressive, hierarchal nature of social emotional ability development within each construct. Next, justification for situating the SEAD model within human development, social, and family theories was presented. Finally, the authors provided a synthesis between constructs of the SEAD model, these theories, and the SCTD that provide explanatory value and insight into the contextual development of social emotional ability (Vygotsky, 1978a, 1978b, 1981).

The first construct, emotional clarity, is comprised of the foundational social emotional abilities to identify, understand, and accept emotions. A review of the literature suggests these social emotional abilities are best defined within the summary construct, emotional clarity. These abilities are progressive in nature because the ability to accept emotions could not fully develop without first having the ability to understand emotions. Furthermore, the ability to understand emotions is dependent upon first attaining the ability to identify emotions.

The second construct, emotional integration, is comprised of the more complex abilities to attend to (i.e., interpret or listen to) emotion, integrate these interpretations into decision-making processes, and regulate emotions. These abilities are more complex in that they progressively integrate cognition, emotion, and behavior. Linkages from the literature were provided which suggest that these abilities are best defined within the summary construct of emotional integration. These abilities are generally progressive in nature because the ability to regulate emotions could not fully develop without first having the ability to respond to emotions; and the ability to respond to emotions is dependent upon the ability to attend to and successfully integrate emotional meaning into adaptive behavioral decisions.

The third and highest ordered summary construct, social integration, is defined by the progressively more complex and abstract ability to respond sympathetically to others with the goal of helping that person to feel better, and the even more complex and abstract ability to empathetically perceive others’ “internal states” and to respond to them through a wide variety of complex and abstract emotionally-based behaviors. Sympathy and empathy represent ways of connecting with others through prosocial response behaviors, such as expressing concern, which underscores the fact that individuals are generally attracted to those that express genuine concern for them (Gottman et al., 1997). Furthermore, prosocial sympathetic and empathetic responses have been shown to be responsible for most healthy human social interaction (Fischer & Manstead, 2016; Keltner et al., 2016; Singer & Lamm, 2009).

### Unique integrations of the SEAD model with existing theory

The authors posit that the SEAD model is novel in its approach to existing human development, social, sociocultural, emotional, and family theory for the following five reasons:

1. The SEAD model posits that social emotional ability is both developmental and hierarchical in nature as scaffolded by bidirectional influences between lower order and higher order ability levels which influence overall ontogenetic development.
2. The SEAD model assumptions are integrated with human development theory which provides structure and support to help explain the continuous, gradual, sequential, and predictable age and stage development of social emotional ability.
3. The SEAD model assumptions are integrated with social and family theory which help to explain the contextual and relational development of social emotional ability.
4. The SEAD model provides the emotional and sociocultural learning context for identifying how social emotional abilities are socialized across cultures, including understanding individual, within-group, and between-group similarities and differences.
5. The SEAD model provides an explanation and conceptualization for how social emotional ability as abstracted from social and emotional intelligence can be condensed to practical skills with broad utility to improve human intrapersonal and interpersonal interaction, thereby improving life satisfaction and well-being.

## Implications

There is an emotional component to all social interaction across all ages and stages of human development because emotions are inherently social in nature. An easy-to-understand, practical, fully-developed, and integrated prevention and intervention model of social emotional ability development holds important and far-reaching implications for researchers, practitioners, educators, couples, parents, and individuals. For example, researchers might use the SEAD model as a guide to improve the understanding of specific impacts of low socialization on individual emotional regulation. The SEAD model sheds light on five specific foundational and hierarchical abilities necessary to healthy emotional regulation in conjunction with sympathy and empathy abilities. Researchers might also use the model to more thoroughly investigate ways in which emotional relationships within the family and the family’s emotional climate influence dyadic relationships and their associated satisfaction and well-being trajectories later in life.

For practitioners, the nine dimensions of SEAD could be used as a diagnostic intervention tool to increase awareness and understanding of their bidirectional influences on contexts, such as family backgrounds and childhood experiences, trauma, adaptive or maladaptive individual traits, and learned interactional processes that promote or inhibit healthy social interaction among couple and family relationships. For example, clinical practitioners could begin with diagnostic assessment of emotional clarity and then use this diagnosis to shape the client’s treatment plan through closing needed gaps in social emotional ability levels to identify, understand, and accept their own and other’s emotions.

Additional examples of how each of the nine SEAD model dimensions could be incorporated into clinical intervention strategies include: (1) Cognitive-behavioral therapy—SEAD conceptual dimensions of emotional clarity may provide utility for assisting clients to identify and better understand causes of emotional distress. SEAD conceptual dimensions of emotional integration could also be used to reduce paired associations between felt emotions and dysfunctional coping behaviors (Epstein & Baucom, 2002); (2) Emotionally Focused Therapy—SEAD conceptualizations of emotional clarity and emotional integration could be applied to assist couples with better processing and interpretation of their emotions as well as increasing empowerment toward healthy emotion management and regulation (Greenberg, 2004, 2016); (3) Bullying interventions—SEAD social integration dimensions could be used as a conceptual foundation in conjunction with empathy-based anti-bullying programs, such as Roots of Empathy (2021), to emphasize the importance of achieving foundational and fundamental levels of emotional clarity and integration among children and teenagers with the therapeutic goal of increasing sympathetic and empathetic response behaviors; (4) Parenting interventions—SEAD conceptual dimensions of emotional clarity and integration can assist emotion-coaching parents to focus on helping their children first identify, understand, and accept their emotions before expecting them to attend to, make healthy decisions, and regulate their emotions.

It is important to keep in mind that the nine abilities of the SEAD model are practical and teachable. Therefore, family science and other social science educators might use guidance provided by the SEAD model to create or expand curricula important to student development as well as personal and professional development. For example, the SEAD model could be used by educators as a roadmap for the development of public school curricula, adult education courses, and family and couple relationship workshops. Couples could employ the instrument to identify strengths and weaknesses in specific areas of emotional clarity, emotional integration, and social emotional integration that could help them better modulate emotion, de-escalate conflict, and promote empathic communication. Parents might also use the instrument to help provide direction for childrearing, to teach emotional regulation, sympathy, and empathy skills, and to improve inter-familial and intergenerational interaction. Measurements could also be used by individuals to guide efforts to improve their social emotional ability and thereby improve their social engagement experiences and resultant levels of life satisfaction and well-being. The SEAD model could also prove useful as a tool to help improve interactions between individuals, co-workers, and colleagues across a variety of workplaces.

Because of the intrapersonal and interpersonal nature of human interactions, the SEAD model could also have broad implications for advancing other theories, such as Gardner’s Theory of Multiple Intelligences (1983), which asserts that varying levels of intrapersonal and interpersonal abilities exist that can be measured among diverse individuals and groups. More specifically, because there is an emotional and behavioral component to all self- and others’ communication, the SEAD model could add practical utility to better understanding cognitive appraisals and emotional interpretations within the context of linear and relational, individual and collectivist, and religious and secular worldviews. Thus, application of the SEAD model to studies of social emotional development across cultural, ethnic, and geographical divides is strongly encouraged for future research.

Finally, although not the focus of this treatise, the authors assert that the SEAD model can be integrated with the CASEL (2021) social emotional learning (SEL) framework and other interventions (Domitrovich et al., 2017; Weissberg et al., 2015) to provide greater awareness surrounding specific developmentally hierarchical skillsets associated with increased social emotional abilities and competencies. More specifically, while the SEL concentric framework introduces five broad constructs of competence (i.e., self-awareness, self-management, social awareness, relationship skills, and responsible decision-making), the SEAD model proposes nine specific developmental, hierarchical skillsets which incorporate bidirectional scaffolding as social emotional learning increases across ages and stages of human development. While the SEL framework is particularly helpful in explaining the contextual influences on social emotional learning specific to a K-12 target audience, the SEAD model employs Vygotsky’s sociocultural learning theory to discuss potential contextual influences on the development of social emotional abilities, including but not limited to the K-12 demographic. Explicit and specific potential associations and integrations between the SEAD model, the SEL framework, and other potential interventions will be addressed in a future treatise.

## Conclusions

The proposed model introduced in this theoretical treatise warrants further investigation for the following reasons: (1) A fully validated SEAD model offers a practical diagnostic tool to identify specific processes inherent to the development of social emotional abilities that can lead to increased levels of healthy and effective social engagement for individuals, families, and couples; (2) The development of a valid, reliable instrument, the Social Emotional Ability Inventory (SEAI), capable of providing diagnostic-level measurements of each dimension of the SEAD model is a logical next step^2^; (3) Construction of such an instrument could provide face validity and empirical construct validity for constructs of the SEAD model; and (4) Measurements provided by subsequent replication studies^3^ could also provide comparison data for triangulation with real-life outcomes for identified stakeholder, safety net provider, and client target audiences.

## Footnotes

1 For purposes of this theoretical treatise, family is defined as “overlapping networks that extend across multiple households, with each network having at its nucleus a reference person” (Amato, 2014).

2 The SEAI instrument has recently been validated and is currently being prepared for publication.

3 A national survey using SEAI is currently in progress.
